# Controllable oscillated spin Hall effect of Bessel beam realized by liquid crystal Pancharatnam-Berry phase elements

**DOI:** 10.1038/s41377-022-00888-4

**Published:** 2022-07-12

**Authors:** Sheng Liu, Shuxia Qi, Yanke Li, Bingyan Wei, Peng Li, Jianlin Zhao

**Affiliations:** Key Laboratory of Light Field Manipulation and Information Acquisition, Ministry of Industry and Information Technology, and Shaanxi Key Laboratory of Optical Information Technology, School of Physical Science and Technology, Northwestern Polytechnical University, Xi’an, 710129 China

**Keywords:** Optical physics, Liquid crystals

## Abstract

Pancharatnam–Berry (PB) phase has become an effective tool to realize the photonic spin Hall effect (PSHE) in recent years, due to its capacity of enhancing the spin-orbit interaction. Various forms of PSHEs have been proposed by tailoring the PB phase of light, however, the propagation trajectory control of the separated spin states has not been reported. In this paper, we realize the oscillated spin-dependent separation by using the well-designed PB phase optical elements based on the transverse-to-longitudinal mapping of Bessel beams. Two typical oscillated PSHEs, i.e., the spin states are circulated and reversed periodically, are experimentally demonstrated with two PB phase elements fabricated with liquid crystal. The displacements and periods of these oscillations can be controlled by changing the transverse vector of the input Bessel beam. The proposed method offers a new degree of freedom to manipulate the spin-dependent separation, and provides technical supports for the application in spin photonics.

## Abstract (web summary)

We demonstrate the oscillated photonic spin Hall effect (corresponding to circular or alternating spin current) by using the well-designed Pancharatnam–Berry phase optical elements fabricated in liquid crystal.

## Graphical abstract

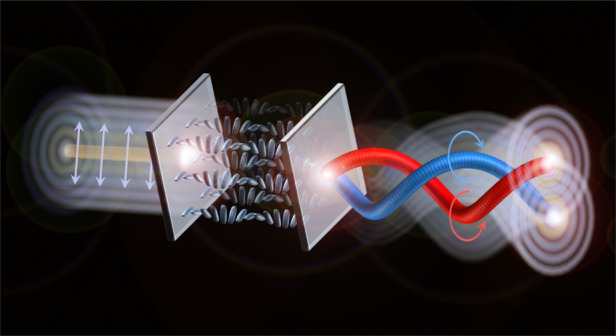

## Introduction

As an analogy of the spin Hall effect in electronic systems, the photonic spin Hall effect (PSHE) has attracted extensive research interests in spinoptics^1^. PSHE is a typical manifestation of spin-orbit interaction (SOI), presenting the separation of the two spin states of light. This spin-dependent separation occurs when the spins change their orientations due to the refraction or reflection at the interface between two different media^2–4^. It is originated from the so-called geometric Rytov–Vladimirskii–Berry (RVB) phase. However, the SOI related to the RVB phase is so weak that the corresponding spin-dependent separation is too tiny to be observed easily, of which the detection generally requires weak measurement technology^4,5^. By means of Pancharatnam–Berry (PB) phase associated with polarization transformation, the SOI of light can be greatly enhanced^6,7^. As such, the strong PSHEs are realized by introducing the PB phase^8–10^. On the other hand, PSHE have been also demonstrated in some particular setups or artificial structures, such as tilted reference frames^11,12^, tilted uniaxial-crystal plates^13,14^, metasurfaces^15^, photonic graphenes^16^, surface plasmon nanostructures^17^, and on-chip devices^18^, et al.

Owing to the SOI induced by PB phase, photonic spin-dependent separations in different manners are proposed prolifically. These are considered as the PHSEs with new styles, and are mainly designed or discovered according to the propagation of spin states^19–24^. For instance, the linear phase gradient generated by the polarization grating leads the two spin states shifting in opposite directions^8^, while the spherical phase leads the spin-dependent focusing^19^. By constructing the PB phase function, researchers have realized the multidimensional manipulation of PSHE^19,23^, the azimuthal^21,22^ and radial^20^ PSHEs, and the location-controlled PSHE^24^. Combined with the phase structures of spatially structured beam, the PB phase can be used to generate spin-controlled Airy-vortex beam^25^ and autofocusing beam^26^. On these bases, many applications, such as optical edge detection^27,28^, PB phase lens^29,30^, photonic spin filter^31^ and sorter^18^ and so on, have been put forward by employing PB phase optical element (PBOE). On the other hand, since the tailoring of PB phase is becoming easier, researchers concern more and more on the controlling of the spin-dependent separation. Although there have been some reports on the controllable PSHE by designing the PB phase^19,23,24,26^, the spin states generally move towards the certain directions. Especially in^24^, an ingenious PB phase was constructed to manipulate the spin separation by shifting the light position. Yet the spin states are still separated in straight lines. How to arbitrarily regulate the spin separation is still a big challenge, because the trajectories of the spin states are difficult to manage only by modulating the PB phase function.

**Fig. 1:**
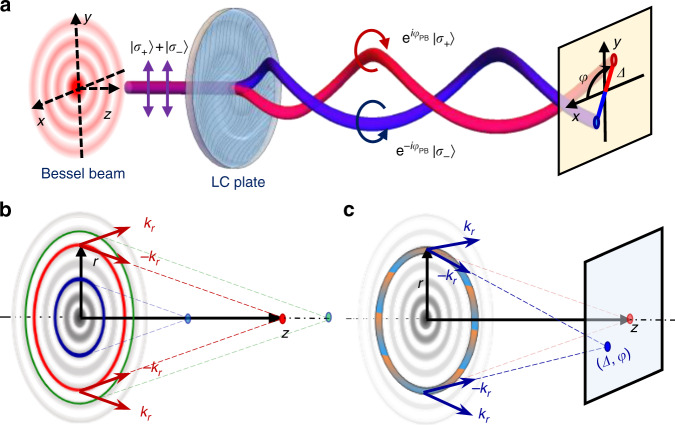
Schematic of oscillated photonic spin Hall effect (PSHE). **a** A linearly polarized Bessel beam passes through a Pancharatnam-Berry phase optical element (PBOE), and splits into two intertwined spin states. **b** Schematic of the transverse-to-longitudinal mapping. **c** Beam deviation under an oblique-phase modulation of a Bessel beam

In this work, we proposed a method to control the PSHE by tailoring the PB phase of Bessel beams, where the trajectories of the separated spin states can be periodically regulated. When a linearly polarized zero-order Bessel beam passes through the PBOE, the circularly polarized components separate from each other and travel along the predefined trajectories, as shown in Fig. 1a. We demonstrate two types of oscillated spin-dependent shifts of Bessel beams via the well-designed liquid crystal (LC) plates, which are fabricated by a dynamic photopatterning technique. Moreover, it is shown that these oscillated PSHE can be also adjusted by the input Bessel beams. The proposed method offers a new degree of freedom to manipulate the spin-dependent separation, and provides technical supports for the application in spin photonics.

## Results

### Controlling propagation trajectory by Pancharatnam-Berry phase

As a typical element for constructing a space-variant polarization or forming a nonuniform wavefront of a beam, PBOE is put forward based on the space-domain PB phase^32,33^. It can be considered as a wave plate with constant retardation and continuously space-varying fast axes, of which the orientation is denoted by *Φ*_PB_/2. Significantly, the PB phase element has different responses for the two orthogonal circular polarization states. Right- and left-handed polarizations (|*σ*_+_〉 and | *σ*_−_〉) are respectively transformed into left-and right-handed ones (|*σ*_+_〉 and | *σ*_−_〉), with attached different PB phases ± *Φ*_PB_, as also illustrated in Fig. 1a. Thus, when a linearly polarized beam passes through the PBOE, the propagation of the two spin states is dominated by ± *Φ*_PB_, respectively. The separation of the spin states during the propagation results in the PSHE.

To control the spin separation, it is ideally to preset the trajectory of the output beams. However, it is difficult to freely manage the propagation path of a beam only by modulating the phase structure. Fortunately, we can get enlightenment from the pre-existing works about the motion analysis and control of the spatially structured beams, such as the self-accelerating beam^34–36^ and the Bessel beam^37,38^. Especially for the Bessel beam, it has shown the controllability in axial intensity and polarization by employing the transverse-to-longitudinal mapping, frozen wave and Gouy phase shift^39–42^. For a zero-order Bessel beam *J*_0_(*k*_*r*_*r*), its propagation can be analyzed by the caustic of rays which focus to form the main lobe. As shown in Fig. 1b, in a circle of radius *r*, there are respectively two sets of rays with transverse wave vectors −*k*_*r*_ and *k*_*r*_ laying on two conical surfaces (red lines). The rays on the wave vector cone −*k*_*r*_ converge to a point on the propagation axis at distance *z*, while the rays of *k*_*r*_ leave away from the axis and contribute less to the main lobe of beam. Namely, the light in the circle *r* will focus to a point on axis at *z*. Similarly, on other circles (blue and green lines in Fig. 1b), the rays converge to different distances on the axis. There is a one-to-one relationship between the radius *r* and distance *z* (see section S1.1, Supporting Information), meeting

1 $$\frac{r}{z} = \frac{{k_r}}{{\sqrt {k_0^2 - k_r^2} }}$$

where *k*_0_ = 2π/*λ* denotes the wave number of light in free space. As such, a transverse-to-longitudinal mapping can be realized: the modulation of the complex amplitude and polarization on the ring of radius *r* will correspondingly change the complex amplitude and polarization on the propagation axis at *z*^39,40^. If a phase gradient is appended in the circle *r*, the focused rays would shift to an off-axis point, forming a spot deviating from the propagation axis, as shown in Fig. 1c. Assuming that the appended phase gradient is expressed by an oblique phase term *Φ* = *k*_*δ*_(cos*ψ x* + sin*ψ y*), and the deviation of the spot is denoted by polar coordinates (*Δ*, *φ*), which denote the quantity and direction of the displacement. It meets $\Delta /z = k_\delta /\sqrt {k_0^2 - k_r^2}$ under the paraxial approximation. Combined with the transverse-to-longitudinal mapping relation, it can be deduced that attaching a radially changed phase gradient *Φ*(*r*) = *k*_*δ*_(*r*) (cos*ψ*(*r*) *x* + sin*ψ*(*r*) *y*) to the input Bessel beam induces the change of propagation trajectory to (*Δ*(*z*), *φ*(*z*)), meeting

2 $$\left\{ {\begin{array}{*{20}{c}} {\Delta \left( z \right) = \frac{{k_\delta \left( r \right)}}{{k_r}}r} \\ {\varphi \left( z \right) = \psi \left( r \right)} \end{array}} \right.$$

where *r* is a function of *z* according to Eq. (1). It should be noted that this equation can only approximately describe the propagation trajectory of the modulated Bessel beam, which might have some difference with the reality, because the transverse-to-longitudinal mapping is not entirely accurate. Only slowly varying trajectory curves are perfectly supported by this method. More details are shown in section S1.2 in Supporting Information.

On this basis, by designing the PBOE with axes along *Φ*(*r*)/2, we can realize the controllable PSHE that two spin states of a Bessel beam separate along the trails (±*Δ*(*z*), *φ*(*z*)), respectively, as shown in Fig. 1a. The signs “+” and “−” denote that the spin’s displacements are in opposite directions, which is attributed to the conjugate PB phases ± *Φ*(*r*) acquired by the two spin states. This event can be also considered as a consequence of SOI: the nonuniform birefringent medium triggers the local wave vector bifurcation of spin states, which indicates the variation of the extrinsic orbital angular momentum related to the spin. The mechanism of spin-orbit interaction is similar to our previous work of analogous optical activity analogy in free space achieved by a PBOE^43^.

### Design and fabrication of Pancharatnam-Berry phase optical element

**Fig. 2:**
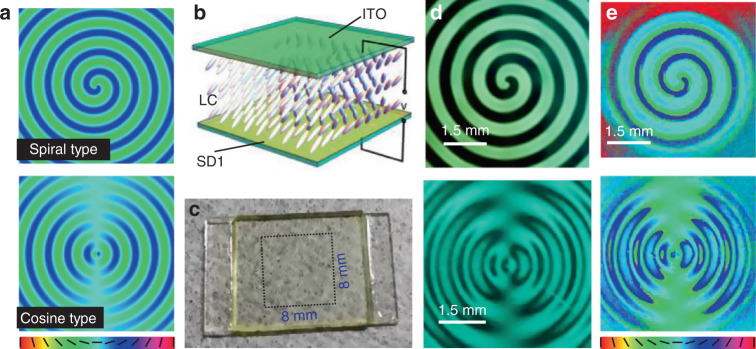
Fabrication of the PBOEs for oscillated PSHE. **a** Predesigned axis distributions of PBOEs. **b** Schematic of liquid crystal (LC) cell. **c**, **d** Photograph and polarizing micrographs of the fabricated LC elements**. e** Measurement results of the axis distributions

To demonstrate the controllable spin shift by the above theory, we design two types of oscillated propagation trajectories, where the direction and quantity of the displacement are periodically varied, respectively. The corresponding coordinates can be respectively described by the parametric functions of spiral line and cosine curve, expressed as

3 $$\left\{{\begin{array}{*{20}{c}} {{\it{\Delta}}\left( z \right) = {\it{\Delta}}_0} \\ {\varphi \left( z \right) = 2{{{\mathrm{\pi}}}}z/{\it{\Lambda}}} \end{array}} \right.\;{{{\mathrm{And}}}}\;\left\{{\begin{array}{*{20}{c}} {{\it{\Delta}}\left( z \right) = {\it{\Delta}}_0\cos \left( {2{{{\mathrm{\pi}}}}z/{\it{\Lambda}}} \right)} \\ {\varphi \left( z \right) = 0} \end{array}} \right.$$

where *Δ*_0_ is a constant denoting the value of displacement, and *Λ* denotes the oscillation period. According to Eqs. (1) and (2), the axis distributions of the corresponding PBOEs are written as

4 $$\frac{{{\it{\Phi }}\left( r \right)}}{2}\left\{ {\begin{array}{*{20}{l}} {\frac{{{\it{\Delta }}_0k_r}}{{2r}}\left[ {\cos \left( K \right)x + \sin \left( K \right)y} \right],} \hfill & {{{{\mathrm{Spiral}}}}\;{{{\mathrm{type}}}}} \hfill \\ {\frac{{{\it{\Delta }}_0k_r}}{{2r}}\cos \left( K \right)x,} \hfill & {{{{\mathrm{Cosine}}}}\;{{{\mathrm{type}}}}} \hfill \end{array}} \right.$$

where $K = 2\pi r\sqrt {k_0^2 - k_r^2} /k_r{\it{\Lambda }}$ (see section S1.1, Supporting Information). By choosing *k*_*r*_ = 3.31 × 10^4^ m^−1^, *λ* = 632.8 nm, *Λ* = 15 cm and *∆*_0_ = 50 µm, the calculated patterns of optical axis distributions of PBOEs are shown in Fig. 2a, where the top and bottom are corresponding to the spiral and cosine types, respectively.

The proposed PBOEs are fabricated in a typical birefringent material LC, which has been widely used to perform the optical field modulation due to its excellent optical anisotropy and electrical tunability. Especially, the LC elements with self-assembled structure or even integrated with metasurfaces introduce new applications in optical steering and switching^33,44–47^. The LC element we used here is a cell of SD1-coated indium-tin-oxide (ITO) glass substrates filled with nematic LC E7, whose optical axis and phase retardation are determined by the azimuthal and polar angles of LC molecules^25,26,43^. Figure 2b schematically exhibits the overall LC director distribution of the designed LC region labeled by the dashed box (about 8 mm square) in Fig. 2c, which is the photograph of the fabricated LC element. A photoalignment technique based on sulfonic azo-dye SD1 and dynamic micro-lithography^33,44^ is employed to guides the LC director according to Eq. (4), and a specific voltage is applied to control the tilt of LC molecules. The detail of the fabrication is given in section S2 in Supporting Information. The corresponding polarizing micrographs are depicted by Fig. 2d, which manifest the optical axis orientation due to the birefringence of LC. The cyanic color of the micrograph is attributed to the corresponding wavelength meeting the half-wave condition. The optical axis distributions of the LC elements are measured by the PB-phase method that we recently proposed^48,49^ by illuminated by a linearly polarized plane wave, as shown in Fig. 2e. The experiment results match well with the pre-designed ones.

### Experiment results

**Fig. 3:**
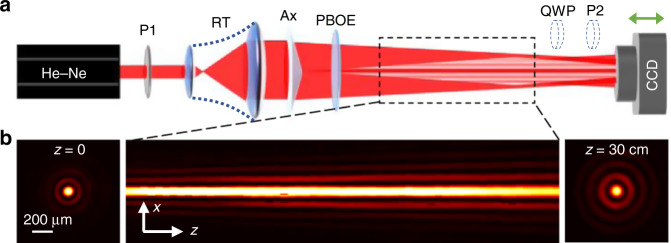
Experimental setup for observing the oscillated PSHE. **a** Schematic of the setup. P, polarizer; RT, reversed telescope; Ax, axicon; QWP, quarter-wave plate. **b** Intensity distributions (left, right) and the side view of the beam propagation (middle) of the generated zero-order Bessel beam

To experimentally demonstrate the proposed oscillated PSHE, we employ the setup as shown in Fig. 3a. The laser beam (He-Ne laser, 633 nm) is expanded by a reversed telescope (RT), and then passes through an axicon (Ax) to generate the input zero-order Bessel beam. The polarizer (P1) guarantees the linear polarization of the input beam. The generated Bessel beam passes through the PBOE and exhibits the oscillated PSHE. The propagation process of the light field is recorded by a CCD camera which moves along the propagation direction step by step. The quarter-wave plate (QWP) and polarizer (P2) can be inserted to separately analyze the left- and right-handed circularly polarized components. Here, we choose an axicon with physical angle 0.5° to match the condition *k*_*r*_ = 3.31 × 10^4^ m^−1^. Figure 3b shows the propagation of the zero-order Bessel beam within a distance of 30 cm, by removing the PBOE. It perfectly represents the non-diffraction propagation with the profile almost keeping unchanged. In consideration of the finite non-diffracting distance from the axicon, the PBOE is put close to the axicon to guarantee a long effective distance as much as possible for observing the PSHE. Notably, since the CCD cannot capture the field on the plane of the PBOE, we start the recording from a certain distance closed to PBOE, which is defined *z* = 0 here.

**Fig. 4:**
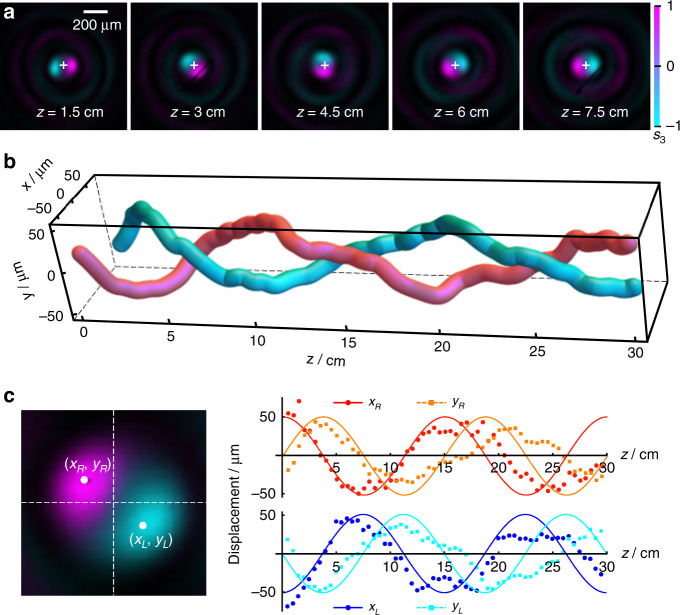
Spiral-like PSHE of Bessel beam. **a** Transverse profiles of the optical fields at different distances, with the background color representing the Stokes parameter *s*_3_. **b** Measured trajectories of the two spin states. **c** Displacements of the spin states vs. propagation distance, where the solid lines depict the theoretical curves

By inserting the spiral-type PBOE shown in top of Fig. 2a, we can observe the spiral-like PSHE. To observe the evolution processes of the two spin states, the combination of P2 and QWP is employed. The two spin components are switched by rotating P2. The experiment results are shown in Fig. 4a, which depicts the beam intensity with the Stokes parameter *s*_3_ represented by the background color. The purple and cyan correspond to the right- and left-handed polarization, respectively. It can be clearly seen that the two spin states of the Bessel beam are separated after passing through the PBOE, and intertwine with each other during their subsequent propagations in free space. It shows a 180° rotation happens within 6 cm propagation. For a quantitative analyzation, the coordinates of the two spin states (*x*_*R*_, *y*_*R*_) and (*x*_*L*_, *y*_*L*_), which are defined as the locations of the peak intensities of the main lobes, are measured step by step. Their trajectories in three-dimensional space and displacements in *x*- and *y*-axes are shown in Fig. 4b and c, respectively. Figure 4b perfectly represents a two-cycle twisting of the two spin states in 30 cm propagation. While in Fig. 4c, it can be clearly seen that the measured displacements manifest the periodic oscillations, similar to the cosine function. The period matches the preset value on the whole. More importantly, the displacements of the two spin states are always opposite, in accordance with the theoretical prediction.

However, there is a discrepancy between the measured and preset values of the displacements. Compared with the predesigned curves in detail, the experiment results only match the theory in the first period. While in the second period, the period in the experiment is larger and the displacement is smaller. This increasing error with propagation distance is mainly resulted from the method of trajectory control, where the error induced by the beam diffraction would be accumulated gradually during the propagation. Either increasing the displacement or decreasing the period of the oscillation trajectory will aggravate the mismatch between the experiment and the predesigned curve. More detailed discussion about the limitation of transverse-to-longitudinal mapping is given in section S1.2 in Supporting Information.

It should be noted that although the experiment results do not match well with the theory, the modulated Bessel beam can still behave the significant oscillating PSHE according to the predefined trajectory. To observe more evident oscillation, we choose a large displacement and a small period in the experiment, which would exceed the tolerance of the slowly varying trajectory curves.

**Fig. 5:**
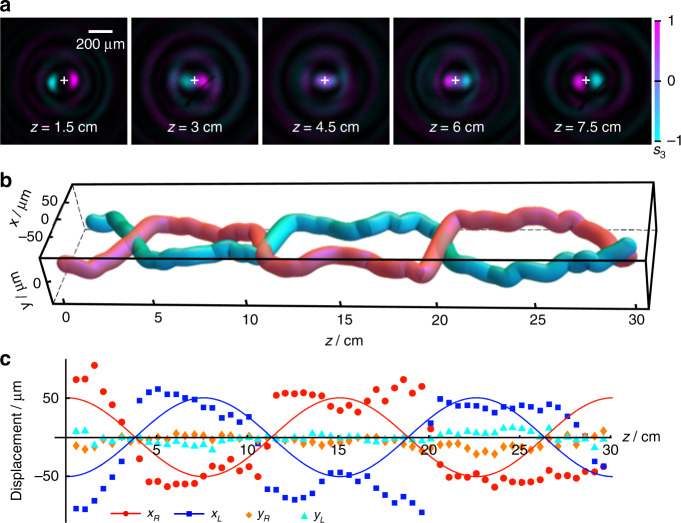
Cosine-like PSHE of Bessel beam. **a** Transverse profiles of the light fields at different distance. **b** Measured trajectories. **c** Displacements of the spin states vs. propagation distance, where the solid lines depict the theoretical curves

To observe the cosine-like PSHE, the PBOE is replaced by the cosine-type one shown in bottom of Fig. 2a. Figure 5a gives the transverse profiles of the modulated Bessel beam in different distances, presenting that the two spin states are first separated, then close to each other, and then separated again in the opposite direction. This oscillated PSHE is visualized by the trajectories in three-dimensional space shown in Fig. 5b, and can be analyzed quantitatively by the displacements of the two spin states shown in Fig. 5c. Yet it is more like a square-wave curve, rather than a cosine curve. This mismatching with the prediction is mainly attributed to the imperfect theory of the transverse-to-longitudinal mapping. However, the oscillated period of the experiment basically coincides with the theoretical value, indicating that the proposed approach is well-suited for the design of the oscillated spin-dependent separation.

### Dependence on the input Bessel beam

**Fig. 6:**
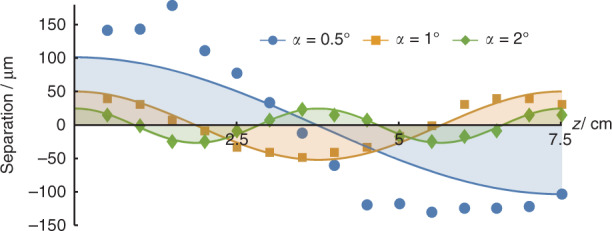
Influence of the input Bessel beam on the spin-dependent oscillated separation. The data points plot the transverse separation between the two spin states of cosine-like PSHE, where the corresponding theoretical curves are depicted by the solid lines. The data for different transverse wave vectors of the input Bessel beams are depicted by blue, orange and green colors, corresponding to the axicons with physical angles *α* = 0.5°, 1°, and 2°, respectively

More strikingly, the proposed oscillated PSHE can be controlled by the input beam, without changing the PBOE. It can be seen from Eq. (2) that for the given phase modulation *Φ*(*r*), the propagation trajectory of the modulated Bessel beam is related to the pre-designed parameter *k*_*r*_. When the transverse wave vector *k*_*r*_ changes, the spin-dependent separations would be also changed. If setting the transverse wave vector of the input Bessel beam as *k*_*R*_, Eq. (4) can be rewritten by replacing *k*_*r*_ to *k*_*R*_, and (*∆*_0_, *Λ*) to (*∆*_0_*k*_*r*_/*k*_*R*_, *Λk*_*r*_/*k*_*R*_) under the paraxial approximation. Namely, the amplitude and period of the oscillated trajectory would be proportionally changed with the ratio *k*_*r*_/*k*_*R*_. To verify this, we change the axicon (the physical angle *α* is selected as 0.5°, 1°, and 2°) to adjust the transverse wave vector of the input Bessel beam, and observe the transverse separation distance between the left- and right-handed spin states of the cosine-like PSHE. The results are shown in Fig. 6, where the solid lines are the corresponding theoretical curves. Obviously, the amplitude and period of the separation distance are decreased by increasing *k*_*r*_, and the decline rate basically accords with the above analyze: when *k*_*r*_ is doubled, the amplitude and period are halved.

In particular, when the transverse wave vector of the input Bessel beam increases, the propagation trajectory of the modulated beam matches the theory more closely, as shown by the orange and green lines in Fig. 6. Although the oscillation of the PSHE becomes faster, the reduce of the displacement seems influence more. The result for *α* = 1° can maintain the coincidence about 5 cm (less than a period), while the distance for *α* = 2° is longer than 7.5 cm (two periods).

## Discussion

The input zero-order Bessel beam in our experiment is generated by an axicon. In addition, the zero-order Bessel beam can also be generated by a LC PBOE with high efficiency and high quality^44^. However, another polarizer will be needed to change the polarization of the generated Bessel beam to a linear one. The integration of the axicon phase profile and the proposed PB phase may provide a further idea to improve the compactness of the whole system. Yet, what we want to verify in this work is that the pure geometrical phase element can directly act on the generated Bessel beam to achieve the oscillated PSHE, which can be also adjusted by changing the input Bessel beam. Thus, a relatively separated but more flexible experiment scheme is adopted.

In the traditional PSHE, the two spin states separate from each other along a certain direction. Here, we demonstrate that the spin-dependent separation can be manifested in more various forms. They can intertwine or cross each other periodically, or even propagate in other different trajectories preset with the proposed method. Especially for the oscillated spin-dependent separation, it reveals that the periodical spin current is formed during the propagation. The spiral- and cosine-like PSHEs represent two different forms of spin currents — circular and alternating spin currents, which can also be considered as an analogy with the electronic system. It opens a new way for the fundamental study in spin photonics.

In conclusion, we theoretically and experimentally demonstrate two typical oscillated PSHEs by tailoring the PB phases of Bessel beams. A linearly polarized Bessel beam passing through the pre-designed PBOE represents spin-dependent separation, with each spin state propagating along a periodical trajectory. The propagation trajectories of the spin states can be controlled by attaching PB phases via the transverse-to-longitudinal mapping of Bessel beam. We fabricate the PBOEs on LC plates by employing the dynamic photopatterning technique. The spiral- and cosine-like PSHEs are experimentally observed by inputting a zero-order Bessel beam generated by an axicon to the PBOEs. The displacement and period of the spin states match well with the theory. Moreover, these oscillated PSHEs can be also controlled by changing the transverse vector of the input Bessel beam. The proposed method offers a new degree of freedom to manipulate the spin-dependent separation, and provides technical supports for the application in spin photonics.

## Materials and Methods

We fabricate the proposed PBOEs in LCs with the photoalignment technique which is based on sulfonic azo-dye SD and dynamic micro-lithography. The LC cell is composed of a pair of SD1-coated indium-tin-oxide glass substrates separated by ~6 μm, and is filled by nematic LC E7. The SD1 is orientated under the illumination of UV light, and guides the LC director by intermolecular interaction. The tilting of LC can be controlled by applying a proper voltage. The details are shown in section S2 in Supporting Information.

## Supplementary information

Supplementary Information

## Data Availability

The data that support the plots within this paper and other finding of this study are available from the corresponding author upon reasonable request.
