# Supplementary material for: Controllable oscillated spin Hall effect of Bessel beam realized by liquid crystal Pancharatnam-Berry phase elements
Source: Light Sci Appl. 2022 Jul 12;11:219. doi: 10.1038/s41377-022-00888-4 (PMC9276670; doi:10.1038/s41377-022-00888-4)
Supplement: Supplementary file 1 — Supplementary Information [file 41377_2022_888_MOESM1_ESM.docx]

Supplementary Information for

**Controllable oscillated spin Hall effect of Bessel beam realized by liquid crystal Pancharatnam-Berry phase elements**

Sheng Liu^#^, Shuxia Qi^#^, Yanke Li, Bingyan Wei^*^, Peng Li^*^, and Jianlin Zhao^*^

*Key Laboratory of light-field manipulation and information acquisition, Ministry of Industry and Information Technology, and Shaanxi Key Laboratory of Optical Information Technology, School of Physical Science and Technology, Northwestern Polytechnical University, Xi'an, 710129, China.*

*# These authors* *contributed equally to this work.*

** Corresponding author:* *wbyxz@nwpu.edu.cn, pengli@nwpu.edu.cn, jlzhao@nwpu.edu.cn*

***Emails of the authors:***

Sheng Liu shengliu@nwpu.edu.cn

Shuxia Qi qishuxia@mail.nwpu.edu.cn

Yanke Li ykli@mail.nwpu.edu.cn

Bingyan Wei wbyxz@nwpu.edu.cn

Peng Li pengli@nwpu.edu.cn

Jianlin Zhao jlzhao@nwpu.edu.cn

1. **Transverse-to-longitudinal mapping of Bessel beam**

**S1.1 Theory of transverse-to-longitudinal mapping**

Theoretically, the establishment of transverse-to-longitudinal mapping comes from the intuitive approach based on the ray theory of geometric optics. According to the angular spectrum theory, a zero-order Bessel beam *J*_0_(*k_r_r*) can be considered as a superposition of a series plane waves, of which the wave vectors are located on a cone as shown in the insert of Fig. S1(b): the radius is *k_r_*, the slant height is *k*_0_=2π/*λ*, and thus the height is *k_z_*=. It can be concluded from a transverse plane of the Bessel beam (Fig. S1(a)) that the fringes of the beam arise from the interference of the rays with transverse vector ±*k_r_*. Here, we only focus on the main lobe of the beam. For the intuitively explanation, we assume that there are a series of concentric rings with radius *r*_1_, *r*_2_, *r*_3_, *r*_4_…*r_n_* on the transverse plane, where each point can launch two rays with transverse vector ±*k_r_*. The ray of *k_r_* on circle *r_n_* converge to a point on the propagation axis at distance *z_n_*, while the ray of -*k_r_* leave far from the main beam region and contribute less to the main lobe. There is a one-to-one relationship between the radius *r_n_* and distance *z_n_*, tan*α*=*r_n_*/*z_n_*, where *α* is the angle between the ray and the propagation axis. In *k*-space (see the insert of Fig. S1(b)), the angle also meets sin*α*=*k_r_*/*k*_0_. Thus, we can get Eq. (1) in the main text.


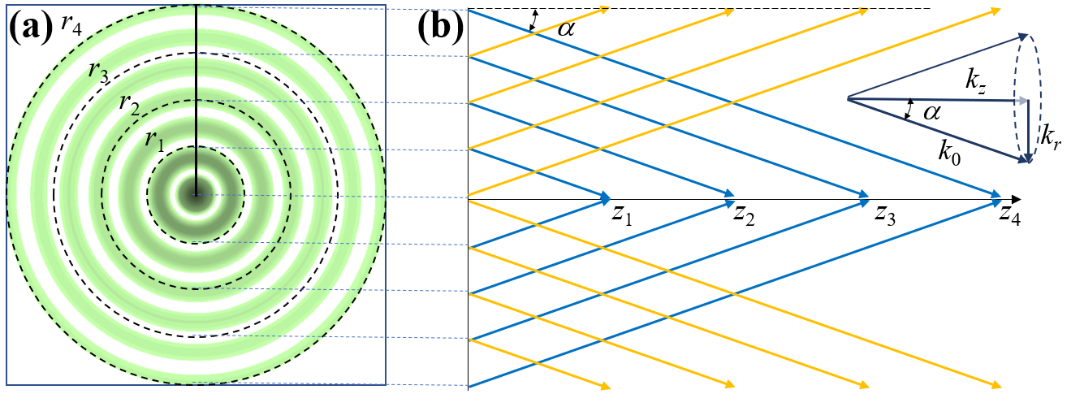


**Figure S1. Schematic of transverse-to-longitudinal mapping of Bessel beam.** (a) Transverse plane of the Bessel beam; (b) Schematic of ray propagation, with the conical wave vector marked in the insert.

Based on the above mapping, the transverse location of the main lobe at any distance *z_n_* can be changed by tilting the wave vector of *r_n_*. Figure S2 visualizes this change. In different rings in the transverse plane, we add different phase gradients to make the wave vector cones titled, and correspondingly the rays focus at off-axis positions. It should be noted that the titling angle cannot be too large, otherwise the rays in a ring would not focus at one position but forms the caustic similar to the cylindrical lens.


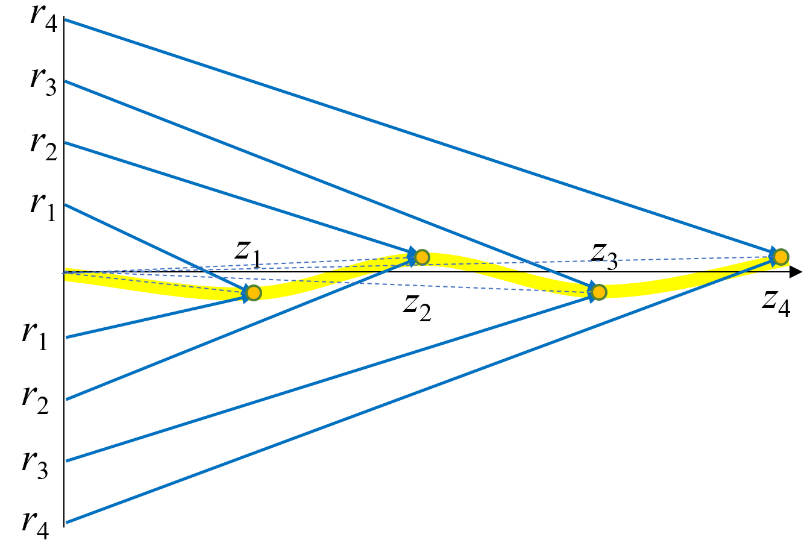


**Figure S2. Schematic of the trajectory control of Bessel beam.**

When giving the appended phase gradient as *k_δ_* (cos*ψ* *x*+sin*ψ* *y*), the displacement of the focusing point of the rays is denoted by polar coordinates (*Δ*, *ψ*), where *ψ* denotes the titling angle, and *Δ* meets *Δ*/*z*=*k_δ_*/*k_z_*. These relationships can be clearly seen from Fig. 1(c). Of cause, we can pre-set the displacement (*Δ*, *ψ*) at *z_n_* firstly, and then design the phase gradient at *r_n_*. Thus, the propagation trajectory (*Δ*(*z*), *ψ*(*z*)) can be designed point by point, and the corresponding phase in the input plane can be calculated by substituting Eq. (1) and *Δ*/*z*=*k_δ_*/*k_z_* to the phase expression, written as

$\Phi\left(r\right)=\frac{\Delta\left(r\right)k_{r}}{r}\left[\cos\psi\left(r\right)x+\sin\psi\left(r\right)y\right]$, (S1)

where (*Δ*(*r*), *ψ*(*r*)) are transformed from (*Δ*(*z*), *ψ*(*z*)) by applying *z*=*rk_z_*/*k_r_*.


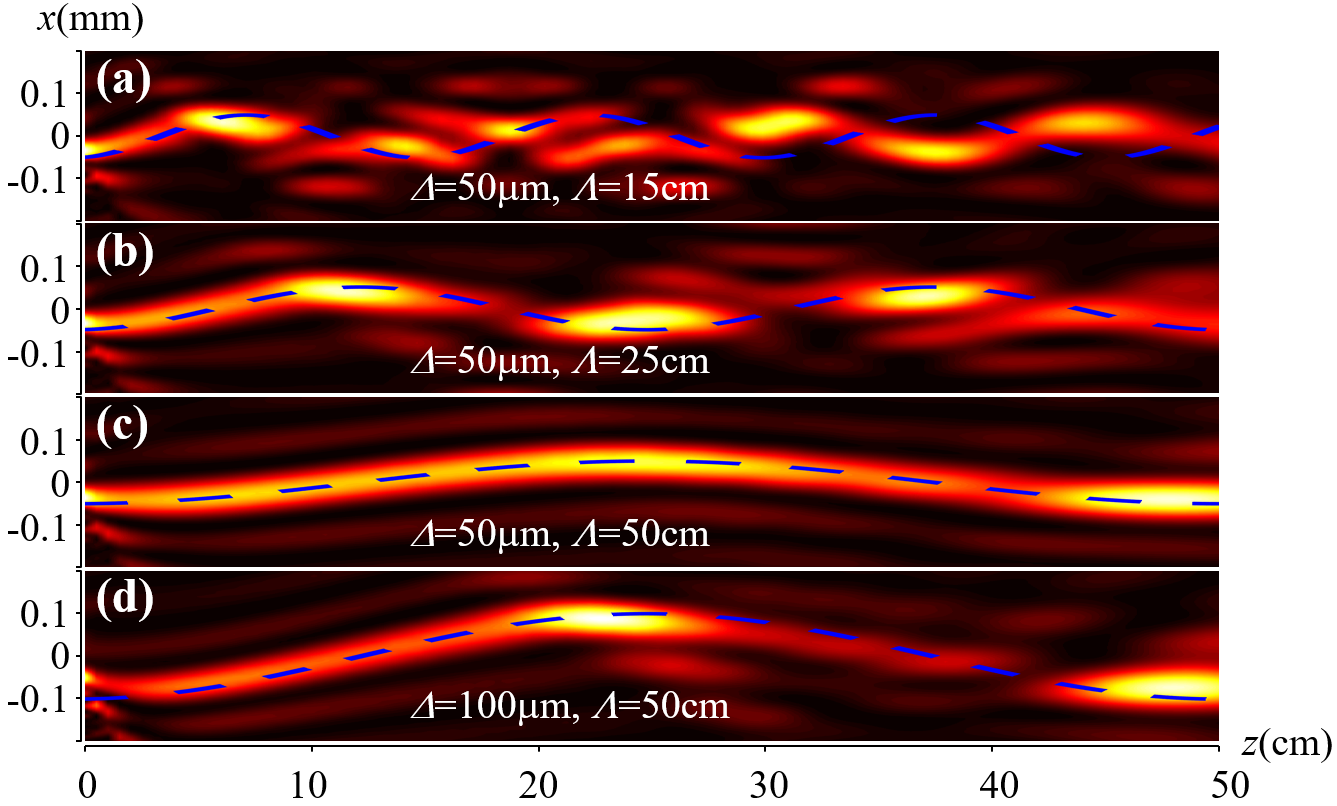


**Figure S3. Influence of amplitude and period on the cosine-like oscillation of the modulated Bessel beam.** Sideview of the propagation processes of the modulated Bessel beams with different *Δ* and *Λ*, where the blue dashed lines mark the predefined trajectories.

**S1.2 Limitation of transverse-to-longitudinal mapping for trajectory control**

Theoretically, the proposed trajectory control method can realize propagation along an arbitrary curve. However, there is a limitation for the transverse-to-longitudinal mapping. As shown in Fig. S2, the titling angle of the wave vector cannot be too large. As a result, the displacement of the beam should be small. Actually, the oscillation period also cannot be small. In a word, if the propagation trajectory is a curve of rapid variation, the propagation of the resulting field would not obey the pre-designed trajectory perfectly, due to the intense diffraction. In Fig. S3, we give the comparation of the cosine-like propagations of the modulated Bessel beams with different parameters. Figures S3(a)-(c) depict the influence of the period of the oscillation trajectory. It is obviously that when the period is large enough (*Λ*=50 cm), the propagation trajectory matches well with the pre-designed curve. When the period decreases to 25 cm, the propagation only matches the curve within ~20 cm. While for the oscillation of period 15 cm (the experimental parameters), only the propagation in a half period conforms to theory. On the other hand, the increase of the oscillation amplitude (see Figs. S3(c) and (d)) also makes the propagation no longer coincide with the theorical curve after a certain propagation distance. As a result, the experiment results both for spiral- and cosine-like photonic spin Hall effects (PSHEs) fail to meet the predefined trajectories.

It should be noted that although the experiment results do not match well with the theory, the modulated Bessel beam can still behave the oscillating propagation according to the predefined trajectory. The linearly polarized Bessel beam passing through the fabricated PBOE can also behave the spiral- or cosine-like PSHE, of which the oscillation period would change after propagating a certain distance. In the experiment, we have chosen the parameters for observing more rapid oscillations of the PSHE, rather than that for coinciding with the theory.

1. **Fabrication of Pancharatnam-Berry phase optical element (PBOE) with liquid crystal**

**2.1 Pancharatnam-Berry phase of the PBOE**

The Pancharatnam-Berry phase optical element (PBOE) is made of a birefringent material with nonuniform fast axes, which is arranged spatially by a specific distribution function *Φ*_PB_(*r*)/2 ^[1]^. Generally, a PBOE has the maximum efficiency when the phase retardation is π. In this case it can be considered as a half wave plate with space-variant fast axis. The transmission matrix of the PBOE is expressed as

$\mathbf{M}\left(r\right)=\left[\begin{matrix} \cos\Phi_{\text{PB}}\left(r\right) & \sin\Phi_{\text{PB}}\left(r\right) \\ \sin\Phi_{\text{PB}}\left(r\right) & -\cos\Phi_{\text{PB}}\left(r\right) \end{matrix}\right]$. (S2)

The PBOE has different responses for the two orthogonal circular polarizations. For an incident wave with circular polarization |**σ_+_**〉 and |**σ_-_**〉, the corresponding output fields are

$\left\{\begin{matrix} |\mathbf{E}_{\text{out}}^{\sigma_{+}}\rangle=\mathbf{M}|\mathbf{\sigma}_{+}\rangle=e^{-\text{i}\Phi_{\text{PB}}}|\mathbf{\sigma}_{-}\rangle\\ |\mathbf{E}_{\text{out}}^{\sigma_{-}}\rangle=\mathbf{M}|\mathbf{\sigma}_{-}\rangle=e^{\text{i}\Phi_{\text{PB}}}|\mathbf{\sigma}_{+}\rangle\end{matrix}\right.$, (S3)

where |***σ*_-_**〉 and |***σ*_+_**〉 are the Jones vectors of left- and right-handed circular polarizations (spin states), expressed as [1, ±i]^T^/. It can be evidently seen that the left and right spins are respectively transformed into right and left ones, and are attached different PB phases exp(±i*Φ*_PB_).

On the other hand, if we want to attached a phase ±*Φ*_PB_ to a left- or right-handed circularly polarized beam, it is a good choice to employ a PBOE of which the fast axis distribution is designed as *Φ*_PB_(*r*)/2. There are generally two requirements for fabricating the PBOE: first, the fast axis of the material is easily arranged according to the pre-designed distribution function; second, the phase retardation is easily managed to satisfy the half-wave condition.

**2.2 Fabrication of liquid crystal plate**

The proposed PBOEs can be fabricated by a typical birefringent material liquid crystal (LC), which has excellent optical anisotropy and electrical tunability. The fabrication of the LC PBOE is carried out by the SD1-based photoalignment technique and the digital micro-mirror device (DMD)-based microlithography system.^[2]^ The SD1 molecules tend to orient their absorption oscillators perpendicularly to the incident polarization direction of the UV light. The orientation of SD1 will further spread to adjacent LC molecules by intermolecular interactions. Thus, we can control the fast axis distribution of LC by spatially changing the SD1 orientation, which is achieved by illuminating the SD1 by a UV light with pre-designed polarization. The DMD system is adopted to realize the precise duplication of designed phase patterns to the polarization of the UV light, as depicted by Eq. (4) in the main text.

A pair of SD1-spin-coated ITO glass substrates, separated by 6 μm, composes a LC cell. After an eighteen-step five-time partly-overlapping dynamic exposure process ^[3]^ and the capillary filling of nematic LC E7 to the cell, an optical axis variant LC PBOE can thus be formed. The phase retardation of the LC PBOE is tunable by applying a proper voltage. Here about 2.5 volts square-wave voltage is applied on the LC to achieve the half-wave condition.

**2.3 Testing of the fabricated PBOE**

The fabricated LC PBOE can be tested by the polarization measurement. We used the PB-phase method

that we recently proposed ^[4]^, as shown in Fig. S4(a). A linearly polarized plane wave [1, 0]^T^ passes through the PBOE to be tested, and changes its polarization into

$\mathbf{E}_{p}=\mathbf{M}\left(r\right)\left[\begin{matrix} 1 \\ 0 \end{matrix}\right]=\left[\begin{matrix} \cos\Phi_{\text{PB}}\left(r\right) & \sin\Phi_{\text{PB}}\left(r\right) \\ \sin\Phi_{\text{PB}}\left(r\right) & -\cos\Phi_{\text{PB}}\left(r\right) \end{matrix}\right]\left[\begin{matrix} 1 \\ 0 \end{matrix}\right]=\left[\begin{matrix} \cos\Phi_{\text{PB}}\left(r\right) \\ \sin\Phi_{\text{PB}}\left(r\right) \end{matrix}\right]$. (S4)

The polarization direction *Φ*_PB_(*r*) is twice the angle of fast axis. Then, the beam interferes with the periodic polarization-structured light field, generates a two-dimensional hologram (interference fringe, see Fig. S4(b)) at the image plane, and then is captured by CCD. From the hologram, the complex amplitudes of the two circularly polarized components (*E_pL_* and *E_pR_*) of **E***_p_* can be simultaneously measured. The angle distribution of the fast axis can be measured by

$\Phi_{\text{PB}}\left(r\right)/2=\arg\left(E_{pL}^{*}E_{pR}\right)/4$. (S5)

Notably that the LC plate might not fit the half-wave condition, i.e. the phase retardation is not π, but *δ*. Thus, the polarization of the beam through the PBOE should be

$\mathbf{E}_{p}=\left[\begin{matrix} \cos\frac{\delta}{2}-\text{i}\sin\frac{\delta}{2}\cos\Phi_{\text{PB}}\left(r\right) \\ -\text{i}\sin\frac{\delta}{2}\sin\Phi_{\text{PB}}\left(r\right) \end{matrix}\right]$. (S6)

In this case, *Φ*_PB_(*r*) is difficult to be solved out directly. Yet, we can use the approximate condition when the PBOE is regulated closely to the half-wave condition. Namely, when *δ*→π, the axis distribution of the PBOE can be expressed approximately by Eq. (S5). The maximum error for |*δ*-π|=0.2π is about 9%.


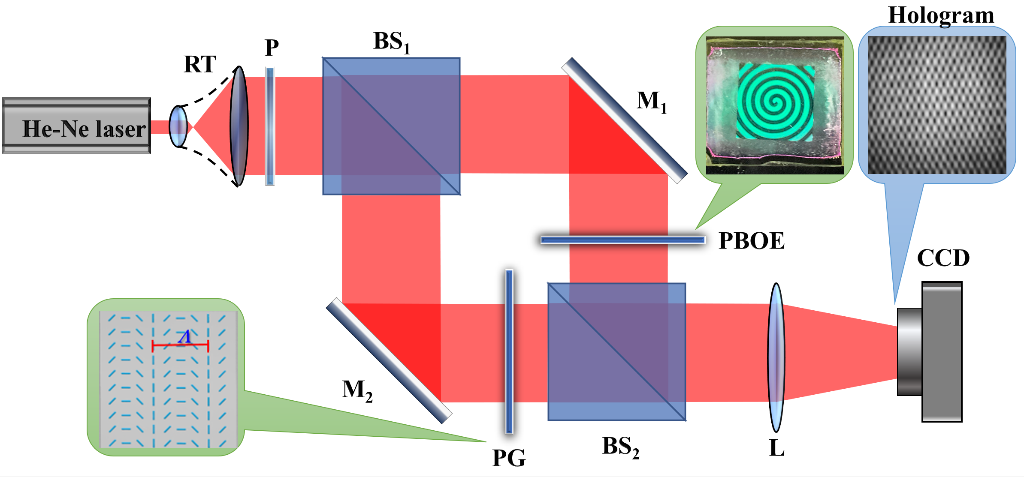


**Figure S4 Setup for testing the fabricated PBOE.** RT: reversed telescope; P: polarizer; BS: beam splitter; M: mirror; L: lens; PG: polarization grating. The PBOE and the PG are imaged by lens L to the CCD plane.

**References**

1. Chen, P., Wei, B. Y., Hu, W. & Lu, Y. Q. Liquid‐crystal‐mediated geometric phase: from transmissive to broadband reflective planar optics. Adv. Mater. 32, 1903665 (2020).
2. Wei, B. Y., Hu W., Ming Y., Xu F., Rubin S, Wang J. G., Chigrinov V., & Lu, Y. Q. Generating switchable and reconfigurable optical vortices via photopatterning of liquid crystals. Adv. Mater., 26, 1590-1595 (2014).
3. Chen, P., Wei, B. Y., Ji W., Ge S. J., Hu W., Xu F., Chigrinov V., & Lu, Y. Q. Arbitrary and reconfigurable optical vortex generation: a high-efficiency technique using director-varying liquid crystal fork gratings. Photon. Res., 3, 133-139 (2015).
4. Qi, S., Liu, S., Han, L. et al. Dynamically measuring the holo-information of light fields in three-dimensional space using a periodic polarization-structured light. Sci. China Phys. Mech. Astron. 64, 264211 (2021).
